# Identification of an IRGP Signature to Predict Prognosis and Immunotherapeutic Efficiency in Bladder Cancer

**DOI:** 10.3389/fmolb.2021.607090

**Published:** 2021-04-15

**Authors:** Liang-Hao Zhang, Long-Qing Li, Yong-Hao Zhan, Zhao-Wei Zhu, Xue-Pei Zhang

**Affiliations:** ^1^Department of Urology, The First Affiliated Hospital of Zhengzhou University, Zhengzhou, China; ^2^Department of Orthopedic Surgery, The First Affiliated Hospital of Zhengzhou University, Zhengzhou, China

**Keywords:** immune-related gene pair, bioinformatic analysis, bladder cancer, TCGA, immunotherapy response

## Abstract

**Background:**

Identify immune-related gene pairs (IRGPs) signature related to the prognosis and immunotherapeutic efficiency for bladder cancer (BLCA) patients.

**Materials and Methods:**

One RNA-seq dataset (The Cancer Genome Atlas Program) and two microarray datasets (GSE13507 and GSE31684) were included in this study. We defined these cohorts as training set to construct IRGPs and one immunotherapy microarray dataset as validation set. Identifying BLCA subclasses based on IRGPs by consensus clustering. The Lasso penalized Cox proportional hazards regression model was used to construct prognostic signature and potential molecular mechanisms were analyzed.

**Results:**

This signature can accurately predict the overall survival of BLCA patients and was verified in the immunotherapy validation set. IRGP-signatures can be used as independent prognostic risk factor in various clinical subgroups. Use the CIBERSORT algorithm to assess the abundance of infiltrating immune cells in each sample, and combine the results of the gene set enrichment analysis of a single sample to explore the differences in the immune microenvironment between IRPG signature groups. According to the results of GSVA, GSEA, and CIBERSORT algorithm, we found that IRGP is strikingly positive correlated with tumor microenvironment (TME) stromal cells infiltration, indicating that the poor prognosis and immunotherapy might be caused partly by enrichment of stromal cells. Finally, the results from the TIDE analysis revealed that IRGP could efficiently predict the response of immunotherapy in BLCA.

**Conclusion:**

The novel IRGP signature has a significant prognostic value for BLCA patients might facilitate personalized for immunotherapy.

## Introduction

Bladder cancer (BLCA) is one of the most common urological malignancies prevalent worldwide. Despite the establishment of several novel treatment strategies, BLCA remains an important medical concern (Bray et al., 2018). According to the latest statistical estimates, there will be approximately 81,400 new cases and 17,980 deaths due to BLCA in the United States in 2020 (Siegel et al., 2020). Once the tumor has developed to a locally advanced or metastatic stage, surgical treatment combined with general chemotherapy is inadequate for the treatment of BLCA (von der Maase et al., 2000; Roberts et al., 2006). There has been considerable progress in research on immune checkpoint therapy, programmed cell death protein (PD-1), and PD-L1 immune checkpoint inhibitors (ICIs), with these therapeutic strategies showing a durable response in advanced BLCA patients (Koshkin and Grivas, 2018). However, these have limited efficacy in most patients; and hence, it is necessary to discover new prognostic biomarkers to closely monitor tumor progression and help classify the patients based on immunotherapy respond.

The tumor microenvironment (TME) is composed of immune cells, stromal cells, extracellular vesicles, and various other molecules. Research reports indicate that the TME is a significant regulator of gene expression and is closely involved in oncogenesis, development, and therapeutic processes (Sharma et al., 2017a; Kamat et al., 2018; van Dijk et al., 2019). More importantly, disorders of immune system processes and immune responses play a crucial role in the TME (Yoshihara et al., 2013). Immune cells can suppress tumor recurrence, progression, and metastasis by interfering with molecular signals and by activating immune responses (Pagès et al., 2005; Yoshihara et al., 2013). Therefore, the uncovering of immune-related gene signatures may help in the stratification of immunotherapy. However, the molecular mechanisms underlying tumor immunity in BLCA remain undetermined, and novel immunotherapeutic stratification markers have not been discovered.

With the development of high-throughput gene detection technology and the establishment of large-scale gene expression data sets, researchers can more accurately identify key molecular features and combine them with clinical features to more accurately stratify patients, thereby develop a personalized treatment plan (Fan et al., 2018; Itzel et al., 2019; Zhou et al., 2019). Previous studies on the development of multiple polygenic signatures based on gene expression signatures can identify high-risk patients. However, due to different platforms for detecting gene expression, the measured gene expression levels are also different. This brings certain difficulties to the comprehensive utilization of these data (Leek et al., 2010). Recently, researchers have provided a new way to solve this problem, namely normalization and scaling based on the relative ranking of gene expression levels. This method has produced reliable results in various studies (Li et al., 2017). Therefore, the purpose of this study is to study the value of immune-related gene pairs (IRGPs) in BLCA, in predicting patient survival, and exploring its potential in predicting the effectiveness of immunotherapy.

## Materials and Methods

### Acquisition of Patient and Sample Data

We downloaded data for three transcriptome cohorts and clinical features (TCGA-BLCA, GSE13507, and GSE31684) from TCGA^1^ and GEO^2^ databases, respectively (Kim et al., 2010; Riester et al., 2012). We set the merged dataset of the three cohorts as the training group. Subsequently, we acquired data for a BLCA patient treated with immunotherapy (EGAS#00001002556) from the European Genome-phenome Archive, and set it as a validation set (Mariathasan et al., 2018). The immunotherapy-treated set contained patient survival information alone, and did not include clinical features. A total of 662 and 195 samples were used in the training and the validation groups, respectively. All samples included data for overall survival (OS) and survival status. As the data used here was obtained from public databases, informed consent was not required.

### Identification of IRGPs in Patients With BLCA

We downloaded the immunity-related gene list (IRG) from the Immunology Database and Analysis Portal (Bhattacharya et al., 2014). The list contains 1811 unique IRGs. We constructed the model using the training set as follows: IRGs were screened out using a median absolute deviation (MAD) >0.5, as they show high variation in all samples. Next, we used the gene expression levels of these genes in each sample for a pairwise comparison to construct IRGPs. In a specific sample, if the expression value of the first IRG was greater than that of the second IRG, the score of the IRGPs in the sample was considered as 1, otherwise it was taken to be 0. The score of each IRGP in all samples was calculated, and the IRGPs with low variation, i.e., with a score of 1 or 0 in more than 80% of samples, were removed.

### Identification of BLCA Subclasses Using IRGPs via Consensus Clustering

The list of IRGPs obtained in the previous step was used for subsequent consensus clustering. Before performing clustering, a filtering procedure was performed. First, candidate IRGPs with a low MAD in all patients with BLCA were excluded. Next, we selected the common IRGPs in the three datasets that constituted the training group. The filtered IRGPs were used for conducting an unsupervised consensus clustering by using the “ConsensusClusterPlus” R package available in Bioconductor. The values of *k* were chosen as the optimal number of clusters based on where the magnitude of the cophenetic correlation coefficient began to fall (Brunet et al., 2004).

### Identification of Prognostic Signature-Based IRGPs

To identify IRGPs for use in clinical settings, a lasso penalized Cox regression (iterations = 1000) was applied using the “glmnet” R package to establish a more stable prognostic model. We screened for IRGPs using 700 repetitions and used the derived coefficients to construct the risk score. A time-dependent receiver operating characteristic (ROC) curve analysis was used to determine the optimal cut-off value for IRGPs in the 3-year OS training group by using the “survivalROC” R package. Based on the cut-off value of IRGPs, patients were divided into high-risk and low-risk groups. The log-rank test was used to evaluate the OS difference between the low-risk and the high-risk groups, and the Kaplan–Meier (KM) survival curve was derived. Further, the ROC curve-based analysis was used to evaluate the sensitivity and specificity of IRGPs. A ROC curve including clinical characteristics was drawn, and the area under the curve (AUC) was calculated. Finally, multivariate Cox regression analysis was used to investigate whether the prognostic value of IRGPs was affected by other clinical characteristics.

### Construction of a Nomogram

The clinical characteristics of the TCGA-BLCA cohort were combined with the IRGP signatures to construct a nomogram by using the “rms” R package. We used the C index to evaluate the discriminative power and draw a calibration chart to evaluate the accuracy of the nomogram. We then compared the decision curve between the clinical characteristics model and the combined model, including the gene signatures and clinical characteristics.

### Gene Set Variation Analysis

Gene Set Variation Analysis (GSVA) is an unsupervised gene set enrichment method that can estimate the scores for certain pathways or markers within a sample population (Hänzelmann et al., 2013). We downloaded the “c2.cp.kegg.v7.1.symbols” gene sets from the Molecular Signatures Database for GSVA. Subsequently, the differential analysis of these gene sets was carried out using the limma package for R software, and the gene set with adjusted *P* < 0.05 was regarded as having differentially expressed genes.

### Evaluation of Immune Cell Infiltration in the TME and Immune Checkpoint Inhibitor Response

The ESTIMATE algorithm was used to calculate the immune and stromal scores which reflect the enrichment of immune and stromal cell gene signatures, respectively (Yoshihara et al., 2013). Additionally, we selected a novel TME-scoring algorithm to explore the differences in TME between the IRGP-based high and low-risk groups (Mariathasan et al., 2018; Zeng et al., 2019). The CIBERSORT algorithm was used to infer the relative proportion of 22 infiltrating immune cells in each sample (Newman et al., 2015). Moreover, the ICI response was assessed by using the tumor immune dysfunction and exclusion (TIDE) algorithm (Jiang et al., 2018).

### Gene Set Enrichment Analysis

The Gene Set Enrichment Analysis (GSEA) software (version 4.0.1) was used to analyze enrichment in high-risk and low-risk groups. The GSEA enriched terms were further analyzed using the Gene Ontology (GO) and Kyoto Encyclopedia of Genes and Genomes (KEGG) databases for high-risk and low-risk groups. A value of *P* < 0.05 and a false discovery rate (FDR) < 0.05 were considered statistically significant.

## Statistical Analysis

All analyses in this study were performed with R software (version 3.6.3) and *P* < 0.05 was considered statistically significant.

## Results

### IRGPs Construction and Consensus Clustering Identifies Two Subclasses in BLCA

**FIGURE 1:**
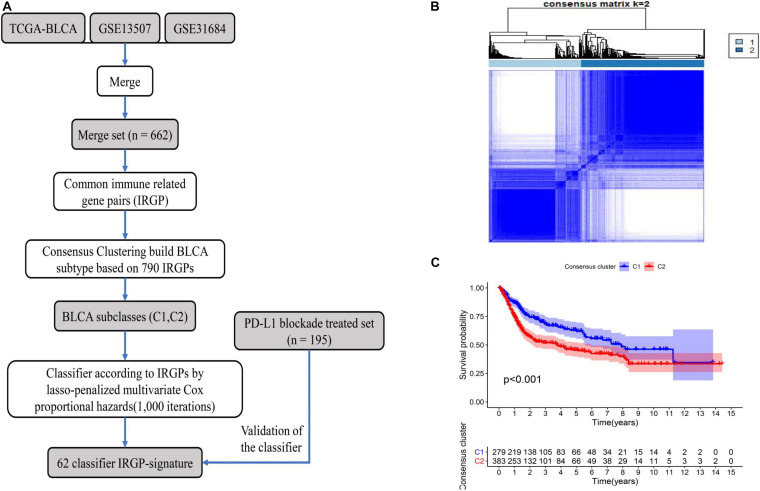
The flowchart describes the construction and validation of our 62 IRGP-signature and consensus clustering of IRGP in BLCA. **(A)** The flowchart about our work; **(B)** consensus matrices of BLCA patients for *k* = 2; **(C)** differences in patient overall survival with two clusters. IRGP, immune-related gene pair; BLCA, bladder cancer.

A flow chart of the entire work is illustrated in Figure 1A. A total of 662 BLCA patients from three cohorts (TCGA-BLCA, GSE13507, and GSE31684) were defined as training set. Through the two-step analysis described in section “Materials and Methods,” 790 IRGPs were constructed by using 432 common immune-related genes, and two subclasses (C1 and C2) were identified based on the 790 IRGPs by using the “ConsensusClusterPlus” package (Figure 1B). In addition, by the log-rank test, the KM curve showed significant survival differences (*P* < 0.001) between the two clusters for OS (Figure 1C).

### Construction and Validation of Prognostic IRGP-Signature

**FIGURE 2:**
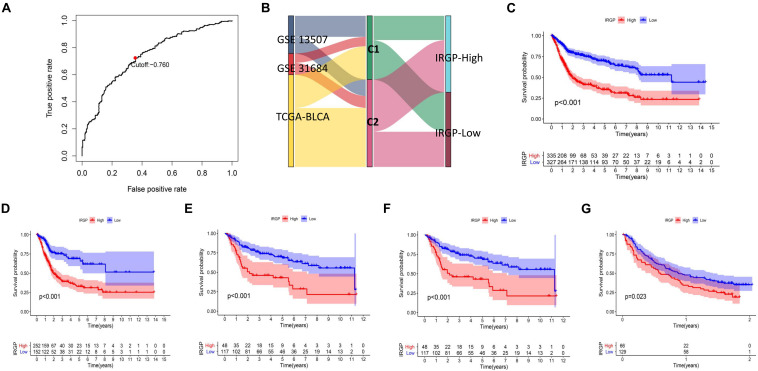
Construction and validation of prognostic IRGP-signature. **(A)** Sankey chart displays the distribution of the consensus clustering in C1, C2, IRGP high-, and low-risk groups. **(B)** Time-dependent ROC curve for IRGP-signature in the training cohort. KM curves of overall survival among different IRGP risk groups in the training set **(C)**, TCGA-BLCA cohort **(D)**, GSE13507 **(E)**, GES31684 **(F)**, and immunotherapy treated validation set **(G)**. IRGP, immune-related gene pair; ROC, receiver operating characteristic; KM, Kaplan–Meier; BLCA, bladder cancer.

Lasso-penalized multivariate Cox proportional hazards modeling was conducted on abovementioned 790 IRGPs. After 1,000 iterations, 62 different IRGP-signature accommodated optimal survival prediction in the training set more than 700 times each. The information of the 62 IRGP-signature is shown in Table 1. Then we calculated the risk score according to IRGP-signature for each patient in the training set. The optimal cut-off of the IRGP between high- or low-risk groups was set at −0.760 using time-dependent ROC curve analysis (Figure 2A). As shown in Figure 2B, the Sankey chart displays the distribution of the consensus clustering in C1, C2, IRGP high-, and low-risk groups. We found that high-risk groups exhibited a significantly poorer OS than the low-risk groups in merged training set, independent TCGA-BLCA, GSE13507, and GSE31684 cohort (Figures 2C–F). Furthermore, in the PD-L1 immunotherapy treated validation cohort (EGAS#00001002556), we used the same cut-off value to divide the patients into high- and low-risk groups. Finally, we can see the high-risk group have a poorer OS (Figure 2G, *P* = 0.023). The IRGP low-risk patients may benefit from immunotherapy.

**TABLE 1:** Information of prognostic IRGP-signature.

IRG1	IRG2	Coefficient	IRG1	IRG2	Coefficient
APOD	CD14	0.16	IL1B	EDNRA	−0.15
APOD	FAM3C	−0.11	IL1B	OASL	0.03
APOD	NR4A2	0.14	IL1B	OLR1	0.09
BPHL	BMP2	−0.04	IL20RA	ICAM2	−0.23
CCR7	CLEC11A	0.21	IL20RA	PTGER4	0.06
CSF1R	IL20RA	−0.04	IRF1	LYN	−0.24
CTSE	FABP4	−0.07	ISG20	CX3CL1	−0.15
CTSE	HLA-DMB	0.03	LCN2	BST2	0.20
CTSE	LMBR1L	−0.52	LCN2	S100A14	−0.17
CTSE	NR2F1	−0.07	LYZ	LTBP2	−0.20
CXCL10	NR2F1	0.25	MICA	ANGPTL4	−0.27
DEFB1	DKK1	−0.32	MMP12	IRF7	−0.16
DKK1	ANGPTL4	0.32	MMP12	MMP9	−0.06
EDN1	ANGPTL2	−0.02	MX1	GBP2	0.13
EDN1	ICAM2	0.04	MX1	TGFBR3	0.33
F2RL1	ANGPTL4	0.06	NFKBIZ	PDGFRB	−0.15
FGR	DKK1	0.10	OASL	IL2RB	0.49
HLA-DMA	IL4R	0.07	OASL	STC1	−0.10
HLA-DMA	SEMA3F	−0.14	PDGFRB	SPP1	0.00
HLA-DPA1	PSMD14	0.13	PLA2G2A	TUBB3	−0.02
HLA-DPA1	SEMA4B	−0.48	PLAU	RARRES3	−0.25
HLA-DQB1	HSPA2	−0.09	PLSCR1	PDGFRB	−0.64
HLA-F	CYR61	0.16	PTGS2	OAS1	−0.26
HLA-F	OAS1	0.36	PTGS2	SYK	0.24
HLA-F	PSMD3	−0.70	S100A14	APOBEC3C	0.11
HLA-G	LCN2	−0.20	SYK	BMP2	−0.22
HMOX1	JAG2	0.44	TINAGL1	PLTP	−0.37
HSPA2	F2RL1	−0.02	TNFSF10	SEMA4B	0.10
HSPA2	NCK1	−0.36	ULBP2	IRF5	0.11
IL1A	EDN1	−0.13	WFDC2	CCL2	−0.08
WNT5A	DCK	0.24	WFDC2	TGFA	−0.12

### Correlation Between IRGP-Signature and Clinical Characteristics

**FIGURE 3:**
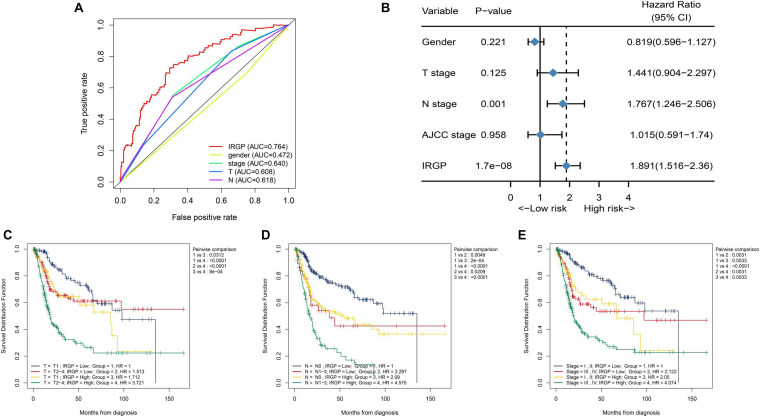
Relationship between risk score and clinical characteristics. **(A)** ROC curve of gender, WHO stage, AJCC-T stage, AJCC-N stage, and risk score with their respective AUC values. **(B)** Multivariate Cox regression analysis. KM curves of OS for patients in TCGA cohort stratified by IRGP signature, AJCC-T stage **(C)**, AJCC-N stage **(D)**, and WHO stage **(E)**. ROC, receiver operating characteristic; WHO, World Health Organization; AJCC, American Joint Committee on Cancer; AUC, area under the curve; KM, Kaplan–Meier; OS, overall survival.

We tend to analyze the correlation between IRGP-signature and clinical characteristics and find which clinical status patients are more suitable for the signature. As shown in Figure 3A, the result of multi-index ROC curve showed that the AUC value of risk score curve (AUC = 0.764) was measured greater than others. We performed multivariate Cox regression analysis to further evaluate the independent prognostic risk factors in TCGA-BLCA set, containing four clinical factors and the IRGP-signature. Then we selected representative three clinical characteristics (AJCC-T stage, AJCC-N stage, and WHO Stage) as the research objects. After analysis in TCGA-BLCA cohort, the results indicated that the OS of patients in the high-risk group was significantly reduced in each subgroup (Figures 3C–E). This result indicates that IRGP-signature may have a powerful prognostic value based on these subgroups.

### Construct and Evaluate Nomogram Based on IRGP-Signature

**FIGURE 4:**
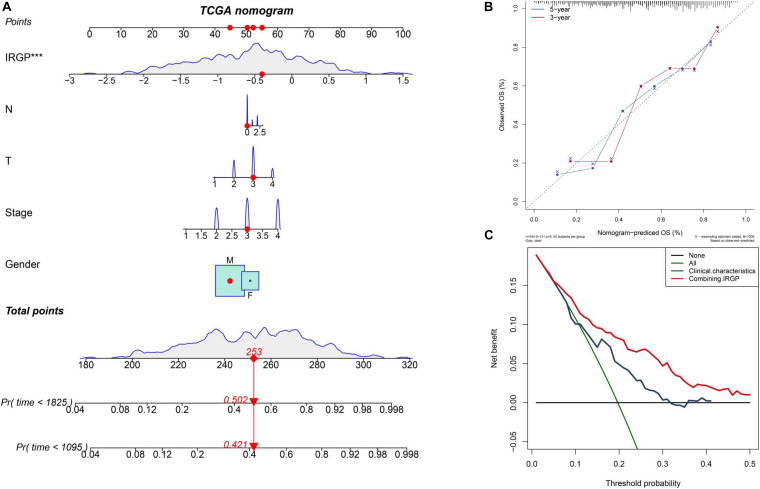
Construct and evaluate nomograms in TCGA-BLCA cohort. **(A)** Nomograms for predicting the probability of patient mortality based on IRPG signature and clinical variables. **(B)** The calibration plot for internal validation of the nomogram. **(C)** Decision curve analyses of the nomograms based on IRGP signature for 3-year overall survival. BLCA, bladder cancer; IRGP, immune-related gene pair.

The nomograms with their clinical characteristics and IRPG-signature were constructed in the TCGA-BLCA cohort respectively. The results of the calibration chart show that the nomogram performance is the best in predicting the 5-year OS. In the TCGA cohort, the C index of the clinical characteristics model and the combined model were 0.697 and 0.903, respectively. The results of the decision curve analysis show that the combined model can bring net benefits in predicting OS (Figures 4A–C).

### Evaluate Differences in TME Between High- and Low-Groups

**FIGURE 5:**
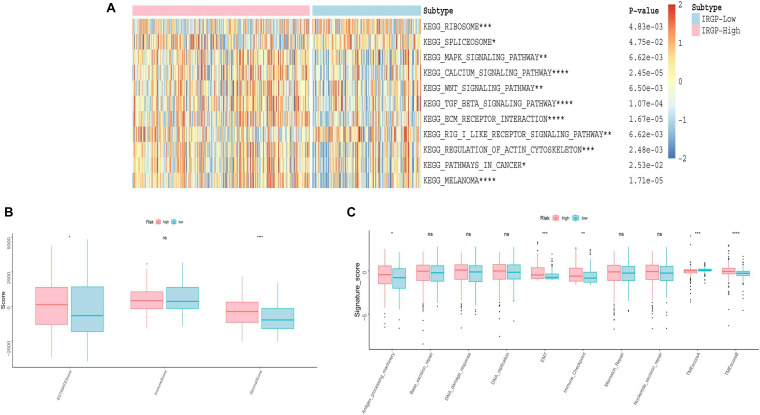
Evaluation of differences in TME between two risk groups. **(A)** Heat map of GSVA analysis between two risk groups. **(B)** Boxplot of immune score and stromal score from ESTIMATE. **(C)** Boxplot of Evaluating novel TME score. EMT, epithelial mesenchymal transition; TME, tumor microenvironment. *P < 0.05, **P < 0.01, ***P < 0.005, ****P < 0.001, ns, no significance.

We used GSVA to explore the differences in biological pathways between the two groups of BLCA patients. As shown in the Figure 5A, we found that in the high-risk group, stromal relevant pathways, included transforming growth factor (TGF)-β signaling pathway and extracellular matrix (ECM) receptor signaling pathway, had higher GSVA scores. Furthermore, ESTIMATE algorithm was used to calculate the immune and stromal score for each patient (Figure 5B). There is a significant difference, high-risk higher than low-risk group, in the stromal score between the two groups (*P* < 0.001). As shown in Figure 5C, the two groups were distinguished by different known TME signatures. We considered IDO1, CD274, HAVCR2, PDCD1, CTLA4, LAG3, and PDCD1LG2 to be immune-checkpoint-relevant transcripts; and VIM, ACTA2, COL4A1, TGFBR2, ZEB1, CLDN3, SMAD9, TWIST1, and TGRB1 to be TGF-β/epithelial mesenchymal transition (EMT) pathway-relevant transcripts. Consistent with the findings, IRGP-high group was notably linked to high stromal relevant signature score (*P* < 0.0001) and was associated with immune checkpoint relevant signature score (*P* < 0.001). The raw data has been uploaded to the supplementary.

### Correlation of the Two Groups With Immune Infiltration

**FIGURE 6:**
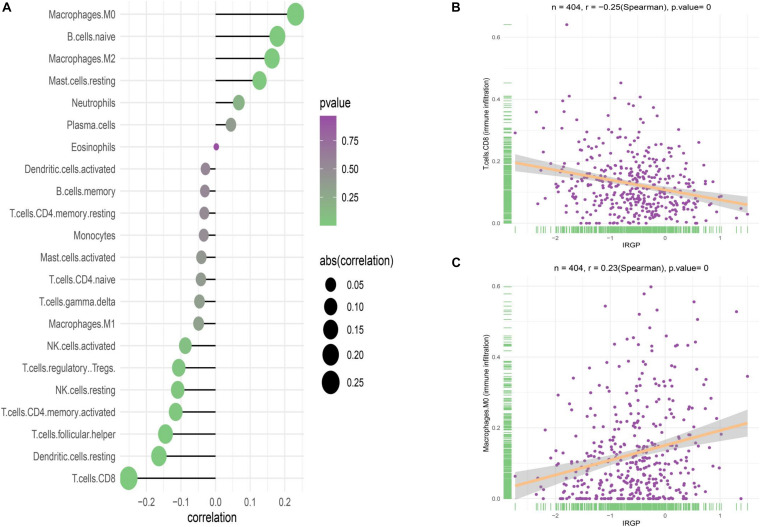
Correlation of the two groups with immune infiltration. **(A)** Correlation of the two groups with 22 immune cells. **(B)** Correlation between CD8+ T cell abundance and IRGP-signature. **(C)** Correlation between macrophages M0 cell abundance and IRGP-signature. IRGP, immune-related gene pair.

Although no obvious difference in immune score was found in ESTIMATE algorithm, we still want to characterize the immunologic landscape of IRGP-high and -low risk groups by investigating immune infiltration. The correlation and abundance of 22 immune-related cell types was calculated using CIBERSORT algorithm and presented in Figure 6 (Gautier et al., 2004). We found the infiltration of CD8+ T cells in low-risk group was greatly higher than that in high-risk group (*P* < 0.001), while macrophages M0 was significantly highly expressed in the high-risk group (*P* < 0.01). The raw data of CIBERSORT algorithm has been uploaded to the supplementary.

### Functional Evaluation of the IRGP-Signature

**FIGURE 7:**
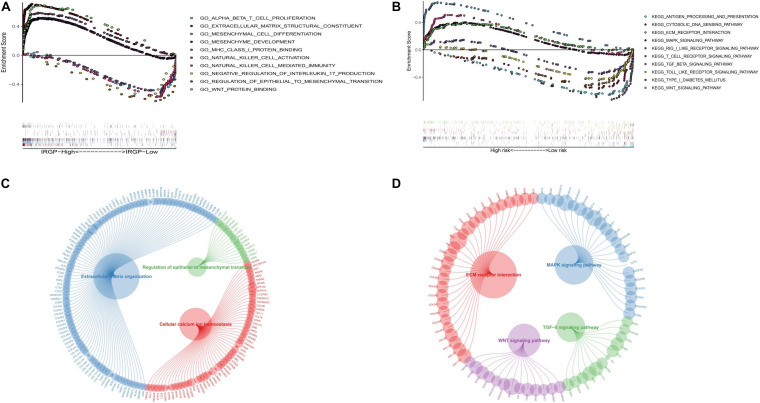
GSEA of the IRGP-signature in BLCA patients. **(A)** The significantly enriched GO terms in TCGA cohort. **(B)** The significantly enriched KEGG pathways in TCGA cohort. Circle plot of gene in GO **(C)** and KEGG **(D)** pathways. GSEA, Gene Set Enrichment Analysis; IRGP, immune-related gene pair; BLCA, bladder cancer; GO, Gene Ontology; KEGG, Kyoto Encyclopedia of Genes and Genomes.

In order to explore the biological processes and signaling pathways changed by IRGP-signature, we performed GO and KEGG analysis by GSEA. Multiple stromal-related pathways, including the ECM signaling pathway, epithelial-mesenchymal transition (EMT) and TGF-β signaling pathway, were highly enriched in high-risk group (*P* < 0.05; Figures 7A,B). Besides, other tumor progression related signaling pathway were also enriched in high-risk group (*P* < 0.05). The circle plot clearly illustrates that the differential genes between high- and low-risk groups contained in these pathways (Figures 7C,D). The heart map displayed the differential expression of specific signaling pathway related genes (Supplementary Figure 1).

### Distinct Sensitivity of Immune-Checkpoint Inhibitors for Two Risk Groups of BLCA

**FIGURE 8:**
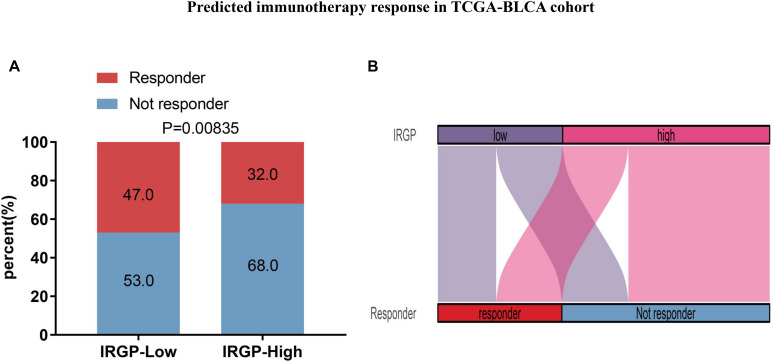
Predicted immunotherapy response in TCGA-BLCA cohort. **(A)** Comparation of immunotherapy responder between two risk groups. **(B)** Sankey chart displays the distribution of responder and IRGP-signature.

Due to IRGP signature can identify patients with better prognosis in the immunotherapy verification set, we next wonder to further verify the stability of the result. The TIDE algorithm, which was established to predict the immunotherapy responders through transcriptomic data, was used to explore whether IRGP could predict immunotherapeutic benefit in TCGA-BLCA cohort. The results of every patients were shown in Supplementary File 4. The output revealed that the percentage of immunotherapy responders were significantly higher in IRGP low-risk patients (47%) compared with IRGP high-risk patients (32%) (Chi-square test, *P* = 0.00835; Figure 8). The IRGP risk-score was robustly negative correlated with the immunotherapy response in BLCA patients.

## Discussion

In China, the mortality and morbidity rates of BLCA are increasing every year (Chen et al., 2016). However, effective treatment strategies for BLCA have not been discovered to date. It is known that lymph node metastasis or muscle invasion by tumor cells affects the treatment result and leads to a poor prognosis (Jacobs et al., 2010). In recent years, immunotherapy has become a high-profile treatment strategy in the area of cancer research due to a better understanding of the association between the immune system and the occurrence and development of tumors. This is evident from the increasing numbers of PD-1/PD-L1 checkpoint blockade agents that have been approved, including pembrolizumab and atezolizumab which display clinical efficacy (Powles et al., 2014; Chabanon et al., 2016; Feld et al., 2019). Thus, the treatment of BLCA patients with advanced disease showing a durable response has become possible (Koshkin and Grivas, 2018). In the past, the expression levels of PD-1/PD-L1 served as an important biomarker to determine the requirement for anti-PD-1/PD-L1 drugs. However, with more immune checkpoints identified, independent PD-1/PD-L1 expression is now considered as an unstable biomarker as there is significant heterogeneity between PD-1/PD-L1 expression and the clinical outcome in BLCA patients (Bellmunt et al., 2014; Massard et al., 2016; Rosenberg et al., 2016; Sharma et al., 2017b). Consequently, attention is shifting to the exploration of novel biomarkers to investigate the potential of personal treatment response to immunotherapy and predicting survival outcomes.

The identification of prognostic signatures by traditional methods requires the pretreatment of gene expression profiles, which is the main factor limiting their widespread use. In this study, we used a merged dataset (TCGA-BLCA, GSE13507, and GSE31684) for training purposes to compare the expression of immune genes from the same sample in pairs. Using this approach, we constructed a signature that can be used widely across different detection platforms (Li et al., 2017). Next, a consensus clustering was conducted using IRGPs, and based on the results we used the lasso-penalized multivariate Cox proportional hazards model to further identify a signature of 62 IRGPs that were most relevant to the prognosis. Based on the signature, patients were divided into high-risk and low-risk groups according to the calculated cut-off value. As shown in Figures 1C, 2C, the IRGP signature can accurately screen patients with a better prognosis as compared to consensus clustering. Based on our results, the IRGP signature, the stable cut-off value verified in the PD-L1 immunotherapy cohort and various clinical subgroups, and a higher IRGP-score together indicate a worse prognosis. It is evident that the IRGP-signature eliminates the heterogeneity between different cohorts to a great extent, and can potentially be used as an immunotherapeutic stratification biomarker for BLCA. Further, we found that combining the signature and clinical characteristics to construct a nomogram can predict the patient’s OS more accurately, and is thus more beneficial.

Scientists usually hope to stratify immunotherapy through an analysis of the differential expression of immune genes. Successful control of tumors by immunotherapy requires the activation of the immune system, the expansion of effector T cells, the infiltration of activated effector T cells into the tumor tissue, and the destruction of tumor cells. However, non-immune cells such as the ECM and stromal cells in TME also play an important role in suppressing or enhancing the immune response (Sun et al., 2018). Therefore, we conducted a comprehensive analysis of the high and low-risk patient groups by estimating the abundance of 22 immune cell types in BLCA and developing a new TME score model, including an immune signature (TMEscoreA), and a stromal activation signature (TMEscoreB). In the high-risk group, GSVA, TME analysis, GO, and KEGG enrichment analysis showed a large amount of stromal cell enrichment, as shown in Figures 4A, 7. In addition, the abundance of CD8+ T cells was higher in the low-risk group and the levels of macrophages M0 was higher in the high-risk group. Previously published reports indicate that the ECM and stromal cells can form a barrier in the tumor and prevent T cells from reaching the TME to destroy the tumor (Brassart-Pasco et al., 2020). Increased CD8+ T cell abundance in TME leads to better immunotherapy efficacy, so patients in the low-risk group have a better response to immunotherapy (Li et al., 2019). Interestingly, our results from the ESTIMATE algorithm showed that there were no differences in the immune scores, but the stromal scores were different. Previous study have already shown that scores related to immune genes in BLCA are inversely proportional to the efficacy of immunotherapy (Cao et al., 2020). From our analysis, it is evident that the difference in survival between the IRGP high and low-risk groups, and the difference in immunotherapy responsiveness may result due to stromal cells. Thus, when exploring for personalized and precise treatment methods using immunotherapy, we cannot consider the role of immune cells during treatment in isolation. In BLCA patients, stromal cells may affect the efficacy of immunotherapy to a greater extent (Zhang et al., 2020). Encouragingly, with the help of the TIDE algorithm, IRGP-based analysis proved to be efficient at predicting the immunotherapeutic response in the TCGA-BLCA cohort. Therefore, these approaches provide new insights for exploring the TME of BLCA in the future.

It should be noted that our research has some limitations. First, as our results have been obtained via bioinformatic analyses, they require further experimental verification using methods such as IHC, IF, qPCR, or flow cytometry to explore the function of the IRGP signatures. In future studies, more attention should be paid to regulating the genes in this signature, in order to help patients with BLCA have a better prognosis. Second, although we used the cohort for immunotherapy as the validation set, the data in the validation set lacked clinical features, and thus, we could not explore the relationship between the efficacy of the immunotherapy and the clinical characteristics. Finally, the signature obtained contains many genes that are not convenient for use in clinical applications. With the further development of bioinformatics technology, future research might identify more accurate signatures for predicting the efficacy of immunotherapy for BLCA.

## Conclusion

In conclusion, IRGP signatures can accurately predict the OS of patients with BLCA, and the combination of signatures with data on clinical characteristics can potentially improve survival benefits in patients. In addition, these signatures may help identify patients who are more likely to benefit from immunotherapy. Infiltration of stromal cells in the TME may be one of the reasons for the poor outcomes obtained during immunotherapy.

## Data Availability Statement

One RNA-seq dataset (The Cancer Genome Atlas Program) and two microarray datasets (GSE13507 and GSE31684) were included in this study.

## Author Contributions

L-HZ collected and analyzed the data and wrote the manuscript. L-QL assisted in collecting the data and participated in the writing. Y-HZ assisted in the design of this study. Z-WZ was responsible for all the integrity of data and the accuracy of data analysis. All authors have thoroughly revised the manuscript.

## Conflict of Interest

The authors declare that the research was conducted in the absence of any commercial or financial relationships that could be construed as a potential conflict of interest.

## Footnotes

1 http://cancergenome.nih.gov/

2 http://www.ncbi.nlm.nih.gov/geo/

## Supplementary Material

Supplementary Figure 1 The heart map displayed the differential expression of specific signaling pathway related genes.

Supplementary File 1 The raw data of ESTIMATE algorithm scores.

Supplementary File 2 The raw data of TME scores.

Supplementary File 3 The raw data of immune cell infiltration CIBERSORT algorithm scores.

Supplementary File 4 The raw data of immune checkpoint inhibitor response (TIDE) algorithm.
