# Supplementary figures and images for: Identification of an IRGP Signature to Predict Prognosis and Immunotherapeutic Efficiency in Bladder Cancer
Source: Front Mol Biosci. 2021 Apr 15;8:607090. doi: 10.3389/fmolb.2021.607090 (PMC8082411; doi:10.3389/fmolb.2021.607090)

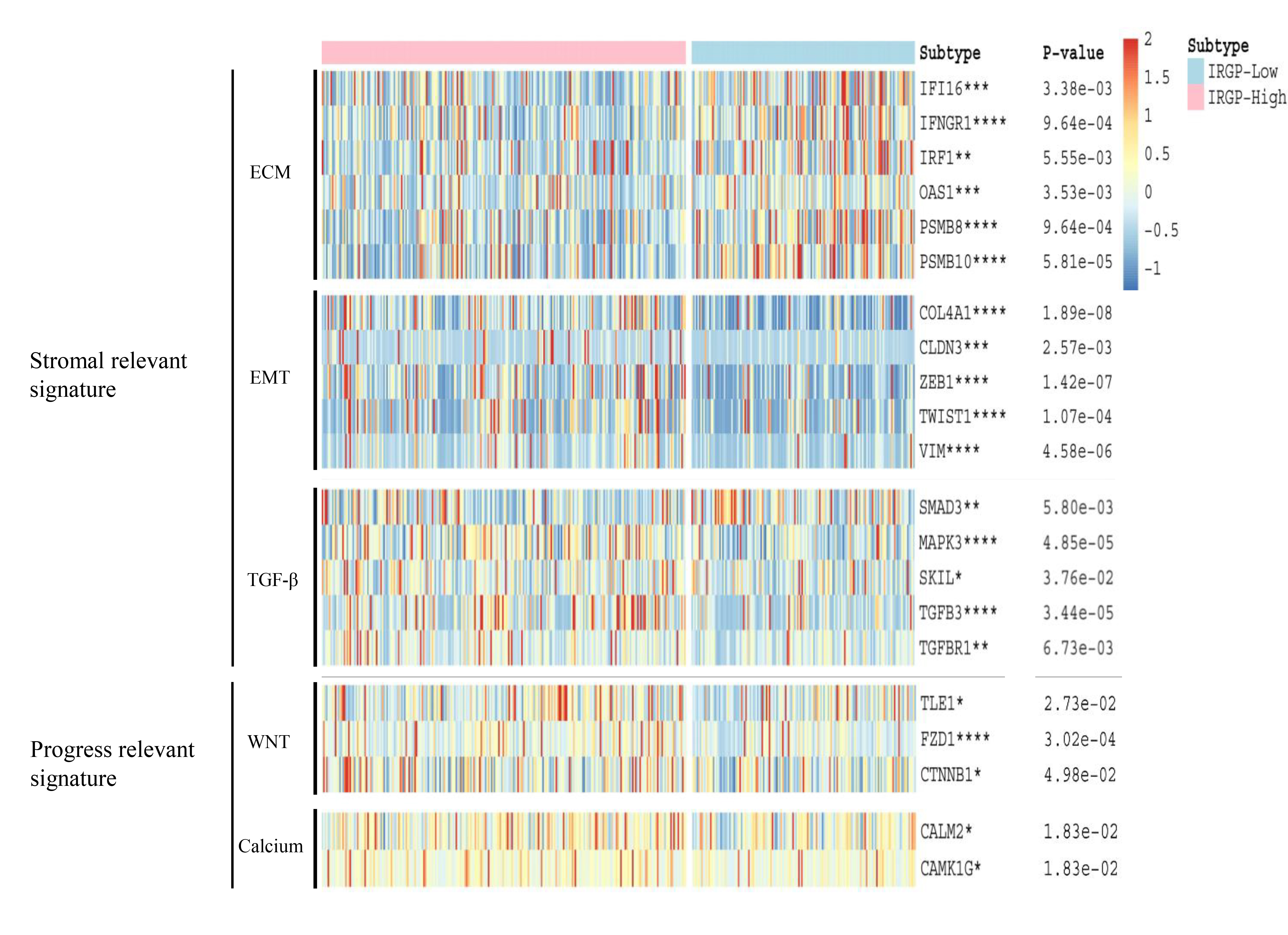

Supplement: Supplementary Figure 1 — The heart map displayed the differential expression of specific signaling pathway related genes. [file Image_1.JPEG]
